# Intense multicycle THz pulse generation from laser-produced nanoplasmas

**DOI:** 10.1038/s41598-023-31427-9

**Published:** 2023-03-14

**Authors:** Manoj Kumar, Hyung Seon Song, Jaeho Lee, Dohyun Park, Hyyong Suk, Min Sup Hur

**Affiliations:** 1 Department of Physics, Ulsan National Institute of Science and Technology, Ulsan, 44919 Republic of Korea; 2 Department of Physics and Photon Science, Gwangju Institute of Science and Technology, Gwangju, 61005 Republic of Korea

**Keywords:** Plasma physics, Laser-produced plasmas

## Abstract

We present a novel scheme to obtain robust, narrowband, and tunable THz emission using a nano-dimensional overdense plasma target, irradiated by two counter-propagating detuned laser pulses. So far, no narrowband THz sources with a field strength of GV/m-level have been reported from laser-solid interaction (mostly half-or single-cycle THz pulses with only broadband frequency spectrum). From two- and three-dimensional particle-in-cell simulations, we find that the strong plasma current generated by the beat ponderomotive force in the colliding region, produces beat-frequency radiation in the THz range. Here we report intense THz pulses $(f\simeq 30 \;$THz) with an unprecedentedly high peak field strength of 11.9 GV/m and spectral width $(\Delta f/f\simeq 5.3\%)$, which leads to a regime of an extremely bright narrowband THz source of TW/cm$^{2}$, suitable for various ambitious applications.

## Introduction

In recent years, terahertz (THz) radiation sources have identified potential in a variety of research and industrial fields including biomedical imaging, time-resolved molecular spectroscopy, communication, and security^1–9^. Despite the rapid advances in THz science today, developing intense and compact THz radiation sources remains challenging. Great strides have been made in crystal-based THz devices^10–15^ that generate sub-millijoule energies of THz pulses with conversion efficiencies reaching around $1\%$. However, it is difficult to scale up to higher energies since the crystals can be damaged at high intensities of driving laser pulses. By contrast, plasma can overcome the problem of optical damage to crystals, because it can sustain extremely high-amplitude electromagnetic oscillations. Such characteristics of plasmas have attracted considerable attention as intense and powerful THz sources^16–20^. Mechanism-wise, ionization current in laser-induced plasma^21–25^, coherent transition radiation (CTR)^26–31^, sheath radiation (SR) associated with energetic ions^32,33^, and experimentally laser-solid target interactions^34–39^generated up to GV/m electric field with 0.1$\%$ conversion efficiency. Recently, structured targets were used to enhance the THz emission by increased laser absorption^40–42^.

The systems described above generate very intense and high-power, but mostly half- or single-cycle THz pulses, which have a broad frequency spectrum. The broadband pulses are useful for ultrafast pump-probe experiments where the THz pulse can be used as a strong DC electric bias field or spectroscopic study of materials^43^. However, spectral density at a particular, desired frequency is inevitably low, hence they are not suitable for applications that require monochromatic THz waves, e.g. THz-driven accelerator^44^ or pump-probe experiments for molecular bonding^45–47^. For plasma-based narrowband THz sources, plasma oscillation^17,19^ can be used as a radiating antenna, but low coupling efficiency between plasma wave and THz radiation matters. Another method is generating THz emission at the beating frequency of two laser pulses that co-propagate through underdense plasmas^20^. In this case, conversion of beat-to-THz is technically difficult and inefficient, and most of all, the plasma current that is responsible for THz emission is weak in underdense plasmas. Other than those ideas, the free-electron laser is only a narrowband source in a high-power regime, but a large facility is required.

This work introduces a novel way to overcome the low plasma current and technical complexity in beat-wave schemes. In our idea, two laser pulses counter-propagate (in contrast to conventional co-propagating schemes) and collide on a nano-dimensional overdense plasma sheet target. As the counter-propagating laser pulses interact with each other on a nano-thickness plasma, the restriction of low-density can be eliminated since the pulses need not propagate inside the plasma. Large plasma current becomes available as the charge carrier density is high in overdense plasmas. No complicated conversion mechanism of beat-to-THz is required; the beat-current can emit the THz wave directly into the vacuum through a thin (below the skin depth) nanoplasma sheet. No plasma shield of electromagnetic emission leads to high conversion efficiency. The number of oscillation cycles in THz pulses can be made arbitrarily large by increasing the duration of driving pulses. From these features, extremely intense multicycle THz pulses with a narrowband frequency spectrum at the beat-frequency of the laser pulses can be obtained with a high efficiency. Our two-dimensional (2D) and three-dimensional (3D) particle-in-cell (PIC) simulations show that such a THz source is capable of providing field strength of GV/m-level, and frequency tunability with a narrowband spectrum. Furthermore, our system preserves the advantage of a single-pulse-driven target, as the broadband THz radiation is emitted together with the desired narrowband one in a single setup.

## Results

### Beat-frequency THz and second-harmonic radiation generation

**Figure 1:**
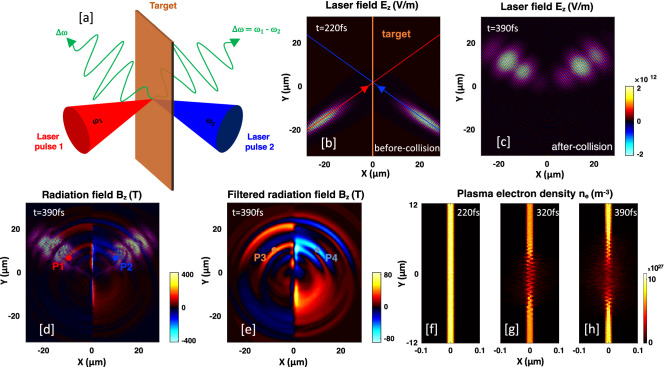
(**a**) Sketch of the scheme: Two laser pulses with different frequencies, $\omega _{1}$ and $\omega _{2}$ collide obliquely with each other on an overdense plasma sheet of nm thickness. From the region of the colliding laser pulses, beat-frequency radiation $(\Delta \omega =\omega _{1}-\omega _{2})$ is emitted into the vacuum. Snapshots of spatial distributions of; (**b**,**c**) laser field $E_{z} (\text {V}/\text {m})$ (s-polarized), (**d**) total radiation field $B_{z} (\text {T})$ (p-polarized), (**e**) filtered radiation field $B_{z} (\text {T}$), and (**f**–**h**) plasma electron number density $n_{e} (\text {m}^{-3})$, at different times; $t=220\; \text {fs}$, $320\; \text {fs}$, and $390\; \text {fs}$.

For 2D PIC simulations, we used the well-known particle-in-cell code EPOCH^48^ (see “Methods” section). The sketch of our scheme is shown in Fig. 1a. Two laser pulses with different frequencies, $\omega _{1}=2\pi c/\lambda _{L1}$ and $\omega _{2}=2\pi c/\lambda _{L2}$, where the wavelengths $\lambda _{L1}=\text {800}$ nm and $\lambda _{L2}=\text {740}$ nm, respectively, and c is the speed of light in vacuum, collide obliquely with each other at an angle $\theta _{\text {col}}=35^\circ$ on a plasma sheet target. The initial thickness and electron density of the plasma sheet are $d=\text {20 nm}$ and $n_{e0}=5n_{c}$, respectively, where $n_{c}=m_{e}\varepsilon _{0}\omega _{1}^{2} /e^{2}$ is the critical density, $\varepsilon _{0}$ is the free space permittivity, $m_{e}$ and $-e$ are the electron mass and charge. The laser pulses are s-polarized (to easily distinguish them from a p-polarized THz emission). The peak value of the normalized vector potential of the lasers is $a_{1,2}=eE_{1,2}/m_{e}c\omega _{1,2}=0.6$ (corresponding to intensities $I_{L1}\simeq 7.7\times 10^{17} \; \text {W}/\text {cm}^{2}$ and $I_{L2} \simeq 9.5\times 10^{17}\; \text {W}/\text {cm}^{2}$), and waist size $b_{w}=5.8 \; \upmu \text {m}$ and pulse duration $\tau _{L}=100 \; \text {fs}$ (FWHM).

As both laser pulses interact with the plasma sheet, their beating exerts a ponderomotive force on the electrons of the plasma sheet that produces a nonlinear current, which emits the beat-frequency radiations $(\Delta \omega =\omega _{1}-\omega _{2})$ in THz frequency range. Along with the THz radiation, the second-harmonics (SH) of the incident lasers are also generated due to the second-harmonic current that is produced by the second-harmonic component of ponderomotive force. The snapshots of the electric field $E_{z}$ (it corresponds to the laser electric field for the s-polarized laser pulses), and the snapshots of the magnetic field $B_{z}$ (it corresponds to the radiation field; the model indicates that it is always p-polarized to the laser incident plane, regardless of the laser polarization) are presented in Fig. 1b,c and 1d, respectively. The source of the emission is clearly at the colliding region of the laser pulses. The low-frequency part of this radiation could be isolated by applying a Gaussian-Filter to the total radiation field (filtered with $f\sim$ 60 THz) as shown in Fig. 1e. As the beat ponderomotive force drives electron oscillations, the plasma electron density is strongly modulated in the colliding region, as shown in Fig. 1f–h. Here we note that when the emitted THz pulses propagate, strong diffraction appears (because the spot size of the colliding laser pulses is smaller than the THz wavelength). The diffraction can be reduced by increasing the laser spot size more than the THz wavelength.

**Figure 2:**
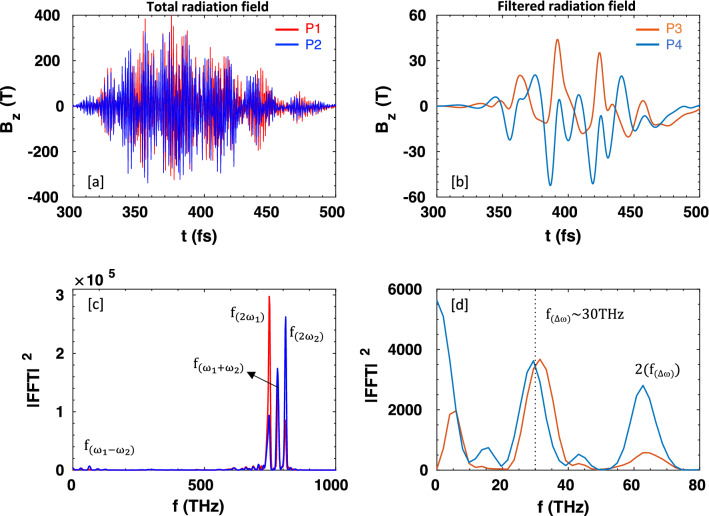
Temporal profiles of (**a**) total emitted radiation, (**b**) filtered beat-frequency radiation in vacuum, measured by the virtual point probes; P1, P2, P3, and P4, located $10\;\upmu \text {m}$ away from the emission spot, and (**c**–**d**) their corresponding power spectra.

To determine the frequency of the emitted radiation in vacuum, we located virtual point probes at P1(*x* = $-10 \; \upmu \text {m}$, $y=7 \; \upmu \text {m}$), P2($x=10\;\upmu \text {m}$, $y=7 \; \upmu \text {m}$), P3(*x* = $-10\;\upmu \text {m}$, $y=10\;\upmu \text {m}$), and P4($x=10\;\upmu \text {m}$, $y=10\;\upmu \text {m}$), to collect the electric and magnetic fields data. The probe positions are denoted by the filled circles in Fig. 1d,e. The temporal evolutions of the total and filtered radiation fields obtained from different probes are shown in Fig. 2a,b. The Fourier spectra of their temporal dependence have the dominant peaks at $2\omega _{1}$, $2\omega _{2}$ (SH of the laser pulses), $\omega _{1}+\omega _{2}$, and a small peak at $\omega _{1}-\omega _{2}$ (Fig. 2c). Here we note that the second harmonic is generated by reflection; $2\omega _{1}$ is dominant on the front (left) side of the target, while $2\omega _{2}$ on the rear (right) side of the target (indicated by the red and blue color lines, respectively). In this study, we focus only on the beat-frequency radiation, so we have removed the high-frequency components from the obtained spectra; Fig. 2d shows a dominant peak near the beat-frequency ($f_{\Delta \omega }\sim 30 \; \text {THz}$) (which represents the emission central frequency), and other smaller peaks at its harmonics $2(f_{\Delta \omega })$. They are obtained from the probe (P3) located on the front-side of the target. Also, the spectrum has a low-frequency peak near $f\sim 5 \; \text {THz}$, which we believe comes through the transition radiation by fast electrons generated from laser-target interactions via different mechanisms^49–51^. A strong direct-current (DC)-like, wideband (0 $\sim$ 10 THz) component is observed from the probe (P4) located on the rear-side of the target (Fig. 2d), which also comes through transition radiation, and comparable strength of the peak at beat-frequency is also found on the rear-side. So, the emitted radiation has a mixture of CTR and beat-frequency ponderomotive force-driven plasma currents. The peak field strengths reach up to $44.2\text {T}$ and $51.4\text {T}$ at $\pm 10\;\upmu \text {m}$ from the sheet surface, which is much higher than other THz sources reported so far.

### Effects of duration and detuning of driving pulses

**Figure 3:**
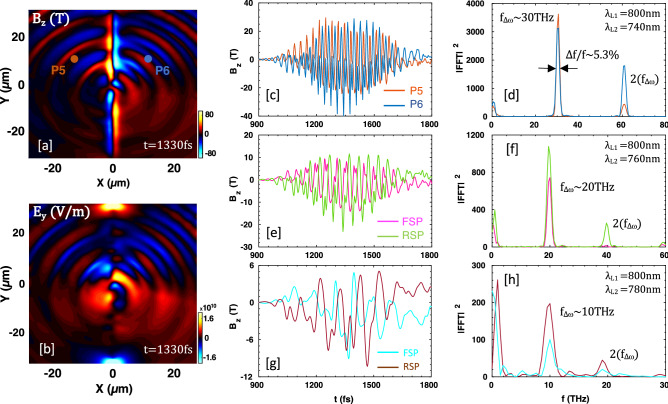
Snapshots of spatial distributions of (**a**) magnetic field $B_{z} (\text {T})$ and (**b**) electric field $E_{y} (\text {V}/\text {m})$ at $t=1330 \; \text {fs}$ for longer pulse duration $\tau _{L}=500 \; \text {fs}$. The field data are filtered to extract the beat-frequency THz range. (**c**) Temporal evolutions of filtered magnetic fields which are acquired from virtual point probes; P5 and P6, and (**d**) their corresponding power spectra. When the wavelength of the second laser pulse is changed, temporal profiles and power spectra; (**e**,**f**) for $\lambda _{L2}=\text {760 nm}$, and (**g**,**h**) for $\lambda _{L2}=\text {780 nm}$.

We now study the cases driven by much longer laser pulses, with duration $\tau _{L}=500\;\text {fs}$ on the same target. Figure 3a shows the strong radiation on the front and back surfaces of the target. Interestingly, in the map of $E_{y}$ (Fig. 3b), strong radiation is found at the top and bottom edges of the sheet, which could be related to antenna-like emission by shielding electron current flowing along the plasma sheet surface^52,53^. Figure 3c shows the temporal profiles of the magnetic field data collected by the point probes; P5(*x* = $-10\; \upmu \text {m}$, $y=10 \; \upmu \text {m}$) and P6($x=10\;\upmu \text {m}$, $y=10\;\upmu \text {m}$), marked by filled orange and light-blue circles in Fig. 3a. Here it is noteworthy that the number of cycles in the radiation pulse has increased in accordance with the increase in pulse duration of the laser. The THz pulse duration is comparable to the laser pulse duration, indicating that THz radiation is generated all through pulse-plasma interaction. Compared to Fig. 2d, the bandwidth of the Fourier spectrum around the beat-frequency ($\sim \; 30\; \text {THz}$) and its second-harmonic ($\sim \; 60\; \text {THz}$) is considerably narrowed, up to $5.3\%$ (Fig. 3d). The peak field strengths reach up to $29.5 \;\text {T}$ ($\simeq \; 8.85\;\text {GV/m}$) and $39.8\;\text {T}$ ($\simeq \; 11.9 \; \text {GV/m}$) on the front and rear sides of the target, respectively. Comparable strength of peak electric field of $10.8 \; \text {GV/m}$ was reported via conventional difference frequency mixing in AgGaS$_{2}$^54^. Here we notice that the second-harmonic of the beat-frequency on the right side is stronger than on the left side of the target. We have not yet identified the reason for that.

We also perform simulations to see the detuning effect of the driving pulses. Figure 3e represents the THz radiation field measured at the same probe positions as before [front-side probe (FSP) and rear-side probe (RSP)], when the wavelength of the second laser pulse is increased to $\lambda _{L2}=\text {760 nm}$ (corresponding to $\sim \; 20 \; \text {THz}$). The radiation field amplitude decreases from the previous case with $\lambda _{L2}=\text {740 nm}$, as the beat-current is proportional to the beat-frequency (actually the linear frequency dependence of radiation amplitude is common for the scheme that uses the plasma electrons as charge carriers). Its power spectrum has a main peak at the beat-frequency ($\sim \; 20 \; \text {THz}$) (Fig. 3f). When the wavelength of the second laser pulse is increased even more up to 780 nm, the THz radiation again peaks at the beat-frequency, i.e. $10 \; \text {THz}$ (Fig. 3h). In the lower beat-frequency cases also, the THz pulse durations are comparable to the driving laser pulse duration (Fig. 3g), implying that by using longer laser pulses (e.g. $\sim \; 1 \; \text {ps}$), the number of cycles in the radiation pulse and the beat-frequency peak value can be increased. Note that the low-frequency radiations near $f \; \sim \; 1 \; \text {THz}$, throughout Fig. 3c–h, do not dramatically change as the second wavelength of the pulse increases, evidencing that the low-frequency radiation comes dominantly from single-laser-target interactions (i.e. CTR) rather than from the beating of lasers.

### Effects of target parameters and collision angle of laser pulses

**Figure 4:**
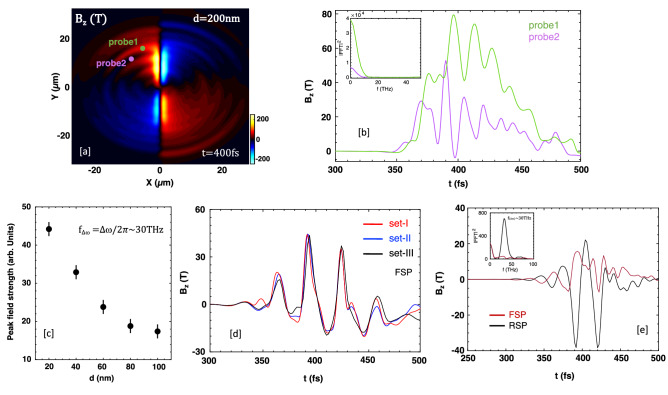
(**a**) Snapshot of spatial distribution of filtered magnetic field $B_{z} (\text {T})$ at $t=400 \; \text {fs}$ for target thickness $d=200 \; \text {nm}$. (**b**) Temporal profiles of emitted radiation measured by; probe1(*x* = $-5\; \upmu \text {m}$, $y=15\;\upmu \text {m}$) and probe2 (*x* = $-10\;\upmu \text {m}$, $y=10\;\upmu \text {m}$). (**c**) Peak field strength of beat-frequency radiation as a function of plasma sheet thickness. (**d**) Temporal profiles of emitted radiation measured by the front-side probe (*x* = $-10 \; \upmu \text {m}$, $y=10 \; \upmu \text {m}$) for three different sets; set-I ($d=20 \; \text {nm}$, $n_{e0}=5n_{c}$), set-II ($d=10 \; \text {nm}$, $n_{e0}=10n_{c}$), and set-III ($d=2 \; \text {nm}$, $n_{e0}=50n_{c}$) from a preloaded plasma sheet target. (**e**) Temporal profiles of filtered radiation fields (measured by the front-side and rear-side probes located $10 \; \upmu \text {m}$ away from the emission spot) from an unionized target, like a graphene sheet, and their corresponding power spectra.

We now examine the effect of the plasma sheet thickness on beat-frequency THz radiation. Figure 4a shows the snapshot of the filtered radiation field for thickness $d=200 \; \text {nm}$, with other parameters same as in Fig. 2. Differently from a thin target ($d=20 \; \text {nm}$), the radiation fills the whole simulation domain (by contrast, upward radiation is dominant in Fig. 1e). Note that, it has different polarities at the front and back surfaces of the sheet. Radiation emission to the surface-normal direction is very small, i.e. conical emission in 3D space, typical of CTR. The temporal profiles of emitted radiation in vacuum and their spectra are illustrated in Fig. 4b. From a thick target ($d=200 \; \text {nm}$), only the half-cycle, low-frequency radiation is generated; no beat-frequency radiation is observed. This is because the target is thicker than the skin depth, so the laser pulses cannot beat with each other. The target interacts with single pulses from either side and the radiation can be produced via CTR mechanism only.

Figure 4c shows the peak field strength of beat-frequency THz radiation decreases with increasing the thickness of the plasma sheet. We also performed the simulations preserving the surface density to 100 (nm$\times n_{c}$) for the sets; set-I, set-II, and set-III, and other parameters are the same as in Fig. 2. The temporal profiles of the filtered radiation field (measured at the front-side of the target only) are illustrated in Fig. 4d. We find that the beat-frequency emitted radiation changes only slightly for different thicknesses but with the same surface density. This result, along with the insensitivity to the density profile of the target, can be a great advantage in designing future experiments; one may use a free-diffused target starting from the ablation of high-density, but thin targets. In this case, THz emission may be insensitive to the delay between the ablating pulse and driver pulses. Another is the use of a graphene sheet target with thickness $\sim$ 2 nm and density $\sim$ 50 $n_{c}$, which is in the neutral state before being ionized. Furthermore, our 2D simulation results also suggest that this system can produce beat-frequency THz radiation with high-peak field strength on the rear-side of the sheet (Fig. 4e), but the front-side emission is significantly reduced compared to a pre-ionized plasma sheet target of the same thickness (see Fig. 4d: set-III]. We have not yet figured out the reason for that.

**Figure 5:**
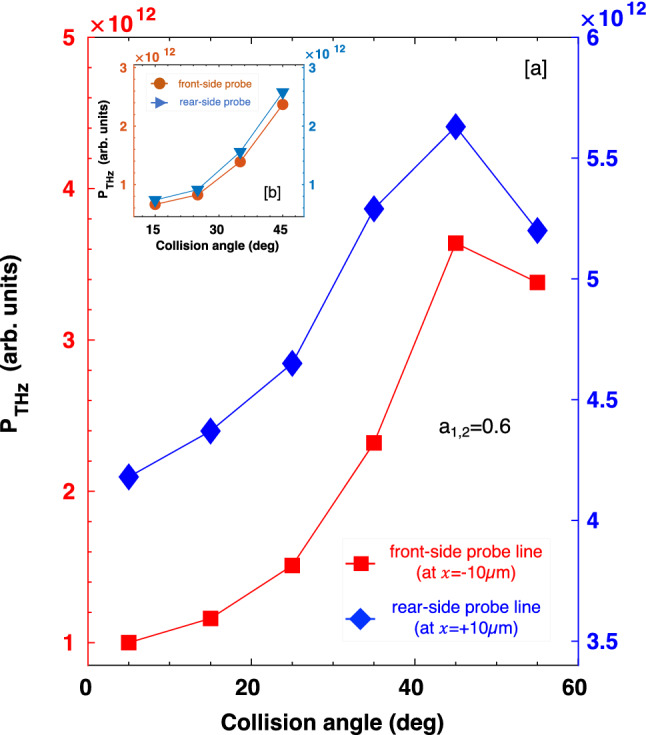
(**a**) Power of THz emission vs. colliding angles of the laser pulses. The power (including the contribution of non-beat-frequency components) is obtained by integrating THz intensity over *y* (from $-32 \; \upmu \text {m}$ through $+32\;\upmu \text {m}$) passing through the probe lines at $x=\pm 10 \; \upmu \text {m}$. (**b**) Power of THz emission with beat-frequency components only.

Now we see the behavior of THz radiation yield for different colliding angles of the laser pulses. The average Poynting flux ($<\text {S}_{\text {rad}}>=(c/2\mu _{0})|B_{0}|^{2}$, $B_{0}$ is the peak value of magnetic field $B_{z}$ and $\mu _{0}$ is the free space permeability) passing through the probing planes (actually probing lines in the 2D domain) at $x=\pm \; 10 \; \upmu \text {m}$ (both left and right sides of the sheet) is integrated over *y* from $y_{1}$ = $-32 \; \upmu \text {m}$, through $y_{2}=+32 \; \upmu \text {m}$, to obtain the power of THz emission. Figure 5a shows that the peak power of THz emission (filtered around $\sim f_{\Delta \omega }$) on both sides of the sheet, increases as the collision angle increases. Because the transverse current is increased for the larger colliding angles, resulting in an enhancement in the field strength of radiation pulses. Although the front-side emission power is lower than the rear-side one, it increases much faster with the collision angles compared to the rear-side emission. This is because the rear-side emission has stronger direct-current components than the front-side one when the colliding angle is small, however, the beat-frequency components are nearly equal on either side of the target and they also have the same emission rate, as shown in Fig. 5b (excluded low-frequency CTR from the emission). When the colliding angle is increased, the direct-current components on the rear-side get weaker, so the rate of emission is also reduced compared to the front-side one. For large collision angle $\theta _{\text {col}}>50^\circ$, the power of THz emission on both sides eventually saturates as the emission is confined near the sheet surfaces.

### Theoretical analysis

In our approach, THz radiation arises from the strong plasma current generated when two laser pulses collide with each other on a plasma sheet. During the interaction, both longitudinal and transverse currents are formed, carried by the plasma electrons. To calculate those currents, the electric field of the laser pulses can be described as follows:

1 $$\begin{aligned} {\textbf{E}}_{1}=\hat{z}\ A_{1}e^{-i[\omega _{1}t-(k_{1x}x+k_{1y}y)]}, {\textbf{E}}_{2}=\hat{z}\ A_{2}e^{-i[\omega _{2}t+(k_{2x}x-k_{2y}y)]}, \end{aligned}$$

where $k_{1x}=(\omega _{1}/c)\cos {\theta _{\text {col}}}$, $k_{1y}=(\omega _{1}/c)\sin {\theta _{\text {col}}}$ and $k_{2x}=(\omega _{2}/c)\cos {\theta _{\text {col}}}$, $k_{2y}=(\omega _{2}/c)\sin {\theta _{\text {col}}}$ are the wavenumbers of the first and second laser pulses and $\theta _{\text {col}}$ is their collision angle. The beat-frequency $(\Delta \omega =\omega _{1}-\omega _{2})$ ponderomotive force (PF) on plasma electrons is given by:

2 $$\begin{aligned} {\textbf{F}}_{\text {P}(\Delta \omega )}=-\frac{e^2({\textbf{k}}_{1}-{\textbf{k}}_{2})}{2mi\omega _{1}\omega _{2}}{\textbf{E}}_1{.\textbf{E}}_2^*, \end{aligned}$$

where *m* and $-e$ are the electron mass and charge. The current density is given by, ${\textbf{J}}_{(\Delta \omega )}=-en_{0}{\mathbf {\textbf{v}}}_{(\Delta \omega )}$, (where $n_{0}$ is the plasma electron density and ${{\textbf{v}}}_{(\Delta \omega )}$ is the plasma electron velocity due to PF), which lies in the $x-y$ plane, so it has two components:

3 $$\begin{aligned} J_{x}= &\,\text {j}_{\text {s}}\left( \frac{\omega _{1}+\omega _{2}}{\Delta \omega }\right) \cos {\theta _{\text {col}}}e^{-i\left[ (\Delta \omega )t-((k_{1x}+k_{2x})x+(k_{1y}-k_{2y})y)\right] }, \end{aligned}$$

4 $$\begin{aligned} J_{y}= &\,\text {j}_{\text {s}}\left( \frac{\omega _{1}-\omega _{2}}{\Delta \omega }\right) \sin {\theta _{\text {col}}}e^{-i\left[ (\Delta \omega )t-((k_{1x}+k_{2x})x+(k_{1y}-k_{2y})y)\right] }, \end{aligned}$$

where $\text {j}_{\text {s}}=a_{1}a_{2}^*m\varepsilon _{0}c^{2}\omega _{p}^{2}/{2e}$, $\omega _{p}^{2}=n_{0}e^{2}/m\varepsilon _{0}$, is the plasma frequency, $\varepsilon _{0}$ is the free space permittivity, $a_{1}=eA_{1}/m\omega _{1}c$ and $a_{2}^*=eA_{2}^*/m\omega _{2}c$ are the normalized amplitude of the lasers.

To estimate the radiated power, we assumed the target thickness is much smaller than the THz wavelength. Therefore, in this limit, the nonlinear current source can be treated like a wire antenna as far as the radiation field is concerned^55^. The total radiated THz power can be described by; $\text {P}_{\text {THz}}=\int _{0}^{2\pi }\int _{0}^{\pi }{{\textbf{S}}_{\text {avg}}r^2\sin {\theta }d\theta d\Phi }$, where ${\textbf{S}}_{\text {avg}}=\hat{r}|B|^{2}c/2\mu _{0}$ is the time average Poynting’s vector, $\mu _{0}$ is the free space permeability, and magnetic field of beat wave is $\textbf{B}=\nabla \times \textbf{A}\simeq i(\Delta \omega /c) \hat{r}\times \textbf{A}$, where $\textbf{A}$ is the vector potential at the far point. Thus,

5 $$\begin{aligned} {\text {P}_{\text {THz}}}=\frac{\pi cm^{2}\omega _{p}^{4}\sin ^{2}\theta _{\text {col}}}{2\mu _{0}e^{2}}\left( \frac{a_{1}a_{2}^*}{2}\right) ^{2}\int _{0}^{\pi }{\frac{\sin ^{2}\left[ {\Delta \omega }L(1-\cos {\theta })/2c \right] \sin ^{3}{\theta }I_{0}^{2}}{(1-\cos {\theta })^{2}}d\theta }, \end{aligned}$$

where $I_{0}=\int _{0}^{r_{d}}{J_{0}(\Delta \omega \rho _i \sin {\theta }/c)\rho _i d\rho _i}$, $J_{0}(x)$ is the Bessel function of first kind zero order, *L* and $r_{d}$ are the length and radius of the wire. In the calculation, we have considered the radiation driven by the beat-frequency ponderomotive force only. The above equation predicts that the emission power increases with $\sin ^{2}\theta _{\text {col}}$ for $\theta _{\text {col}} <{90}^\circ$. There is no THz emission for normal incidence $\theta _{\text {col}}=0^\circ$ because there is no transverse current component.

**Figure 6:**
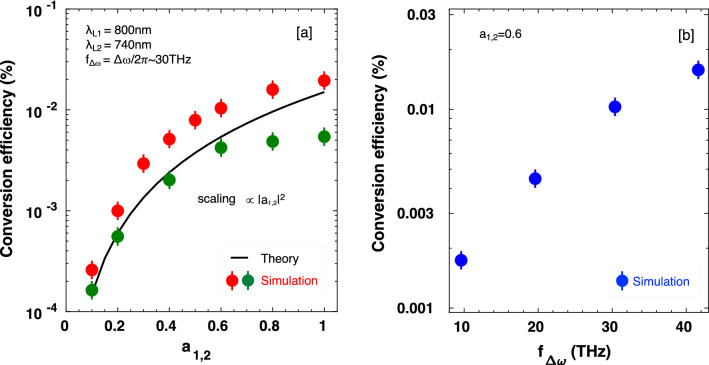
Efficiency of power conversion from lasers-to-THz emissions as a function of; (**a**) normalized vector potential of lasers $a_{1,2}$, (**b**) central-frequency of THz radiation $f_{\Delta \omega }$. The solid-red, -green, and -blue circles are from simulation results (measured at $\pm 10 \; \upmu \text {m}$ from the emission spot), and the solid black line is from theoretical results.

Figure 6a shows the lasers-to-THz power conversion efficiency as a function of the normalized vector potential of lasers, and a comparison of the theoretically obtained efficiency (from Eq. (5)) with simulation results. The efficiency increases with $a_{1,2}$, reaching around $0.02\%$ when the laser intensities are close to $10^{18} \; \text {W}/\text {cm}^{2}$. Note that, if the intensity $>10^{18} \; \text {W}/\text {cm}^{2}$, it begins to saturate presumably due to relativistic effects. Here it can be seen that the simulated efficiency (solid-red-circle) is higher than the theoretical efficiency due to the non-beat-frequency components. When only the beat-frequency components are taken into account in the calculation of the efficiency, the efficiency (solid-green-circle) decreases and approaches the theoretical result. The theoretical scaling is proportional to the square of the normalized vector potential of lasers. Figure 6b shows that the efficiency increases with increasing the beating frequency.

## Three-dimensional PIC simulations

**Figure 7:**
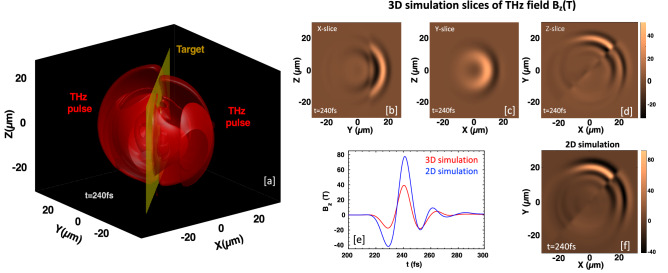
(**a**) 3D isosurface of beat-frequency THz radiation field (in red-color) at $t=240 \; \text {fs}$. (**b**–**d**) 2-D slices (cut through *x*-, *y*-, and *z*-axis) of the THz radiation field. (**e**) Comparison of THz field strength (measured on the front-side of the target) through 2D and 3D PIC simulations for the same set of parameters. (**f**) Snapshot of X–Y spatial distribution of THz radiation field from 2D simulation.

In the previous section, we have dealt only with 2D PIC simulations for the foundation of a novel method supported by theoretical analysis. The realistic model of the proposed system that can be applied to the experiment needs to develop through a three-dimensional simulation study. Therefore, we also performed 3D simulations for a similar situation using EPOCH code. For that we have chosen a target of $56 \; \upmu \text {m}$ wide, $48 \; \upmu \text {m}$ tall, $100 \; \text {nm}$ thick, and has the electron density of $1.0n_{c}$ (to preserve the surface density to $100(\text {nm}\times n_{c})$ same as in our 2D simulations). The target is tilted at an angle of $45^\circ$ with respect to both laser propagation directions. The laser pulses are s-polarized with intensities; $I_{L1}\simeq 7.7\times 10^{17} \; \text {W}/\text {cm}^{2}$ and $I_{L2} \simeq 9.5\times 10^{17} \; \text {W}/\text {cm}^{2}$ (same as in 2D simulation), with $8.4 \; \upmu \text {m}$ spot diameter, but use only a $30 \; \text {fs}$ (FWHM) pulse duration due to limitation by computational resources. The simulation domain is $60 \; \upmu \text {m}$ (in *x*-direction and *y*-direction), and $50 \; \upmu \text {m}$(in *z*-direction), with the grid sizes $dx=dy=dz=\lambda _{L1}/12.5$, and filled with 10 numerical macro-particles per cell. Figure 7a displays an isosurface of filtered beat-frequency THz radiation field (in red-color) at $t=240 \; \text {fs}$. The snapshots of the 2D slices (cut through *x*-, *y*-, and *z*-axis) of the THz radiation field show that the emission originates from the colliding region of laser pulses (Fig. 7b–d). As time goes on, the radiation fills the entire simulation domain, but strong emission is found in the upward region and weak emission in the downward region of the sheet. The emitted THz pulses have one oscillation cycle because their duration is comparable to the laser pulse duration ($\sim$ 30 $\text {fs}$). The peak field strength reaches more than 10 GV/m in the 3D simulation, but it is lower than the 2D simulation result for the same parameters (Fig. 7e). However, the X–Y spatial distribution of the THz field (Fig. 7f) is similar to the 3D simulation result. This strongly indicates that the beat-frequency mechanism will work efficiently for the experiment.

## Conclusion

We proposed a novel scheme to obtain narrowband, readily tunable, compact, and experiment-friendly THz sources based on the oblique collision of two-detuned laser pulses on a nano-dimensional overdense plasma sheet target. The process of THz emission and resulting THz characteristics were investigated by using two- and three-dimensional particle-in-cell simulations. In contrast to single-pulse-driven targets, where long half-cycle THz pulses are generated with a broadband spectrum via CTR mechanism, by irradiating the rear surface of the target with another laser pulse, we produced multicycle, narrowband THz radiation at the beating frequency, dominating over the CTR. The THz radiation was generated due to the ponderomotive force-driven plasma current in the colliding region. We obtained extremely intense THz radiation of 40 $\text {TW}/\text {cm}^{2}$, with peak fields up to $\sim$ 11.9 GV/m and 39.8 T at 10 $\upmu$m from the emission spot, with central frequency $f\sim 30 \; \text {THz}$ and spectral bandwidth $\Delta f/f \simeq 5.3\%$ for $\tau _{L}=500 \; \text {fs}$. The number of cycles of THz oscillation can be increased arbitrarily by increasing the driving pulse duration, which can provide a very narrowband spectrum. By using much longer laser pulses ($\sim$ 1 ps), the spectral bandwidth can be increased up to 3.28$\%$. For the laser intensities $>10^{18} \; \text {W}/\text {cm}^{2}$, the emitted THz pulse energy was about 0.1 mJ. Such an extremely intense narrowband THz source will be suitable for various ambitious applications such as compact electron accelerators^44^ and pump-probe experiments^45–47^. We believe our results will greatly help in designing future experiments on the proposed system.

## Methods

### Simulations code and parameters

The 2D and 3D simulations were performed using fully relativistic particle-in-cell code, EPOCH (Extendable PIC Open Collaboration)^48^. Maxwell’s equations are solved in this code using the finite-difference time-domain (FDTD) method in 2D3V (two-dimensional configuration space and three-dimensional momentum space) geometry. The electric and magnetic fields are specified on Yee staggered grids^56^, and the Boris algorithm^57^ is used for solving the equation of motion of the particles. The currents required for the Maxwell solver are calculated from the particle motion using Esirkepov’s method^58^ which is a generalization of the Villasenor and Buneman current deposition scheme^59^.

In our simulations, both laser pulses are injected into the simulation domain from the left and right boundaries, colliding on the plasma sheet target with different thicknesses and densities. The two-dimensional simulation domain is $56 \; \upmu \text {m}$ long and $64 \; \upmu \text {m}$ wide with a grid size $dx=dy=\lambda _{L1}$/160 in the *x*- and *y*-directions, respectively. Each grid cell is filled with 40 numerical macro-particles (a total of over $10^{6}$ particles in the entire simulation domain). The time step is set to ensure Courant conditions: $\Delta t=0.99 \Delta x/(c\sqrt{2})\simeq 1.667\times 10^{-17}$. For both particles and fields, we use the $\textit{simple\_laser}$ boundary condition at the left and right box boundaries $(x=\mp 28 \; \upmu \text {m})$ and the $\textit{simple\_outflow}$ boundary condition at the top and bottom box boundaries $(y=\pm 32 \; \upmu \text {m})$. We used the BSI (barrier-suppression ionization) model in EPOCH code for the simulation including field ionization.

## Data Availability

The data supporting this study’s findings are available from the corresponding author upon reasonable request.
